# Effect of port-site and intraperitoneal local anesthetic injection versus placebo on postoperative pain and recovery after gynecologic laparoscopic surgery: a randomized controlled trial

**DOI:** 10.1038/s41598-025-18389-w

**Published:** 2025-09-12

**Authors:** Usama A. Elsaeed, Eslam Ahmed Fouad Ismail Hussein Algyoushy, Dina Latif Mahrous Hatem

**Affiliations:** Obstetrics and Gynecology Department, Faculty of Medicine, Cairo University, Cairo, Egypt

**Keywords:** Gynecological laparoscopy, Intraperitoneal anesthesia, Trocar site infiltration, Postoperative pain, Recovery, Randomized controlled trial, Medical research, Clinical trial design, Randomized controlled trials

## Abstract

Laparoscopic gynecological surgery, while minimally invasive, is frequently associated with significant postoperative pain requiring systemic analgesics. Local anesthetic administration, either intraperitoneally or at trocar sites, has been proposed to improve analgesia and recovery, but evidence remains inconsistent. To evaluate and compare the efficacy of intraperitoneal and port-site local anesthetic injection versus placebo in reducing postoperative pain and improving recovery outcomes in women undergoing gynecological laparoscopic surgery. This randomized, double-blind, three-arm controlled trial enrolled 90 women aged 18–60 years undergoing elective gynecological laparoscopy at Kasr Al-Ainy Hospital, Cairo, Egypt (March 2024–March 2025; ClinicalTrials.gov NCT07030647). Participants were randomized into: Group A (trocar site bupivacaine 0.25%), Group B (intraperitoneal bupivacaine 0.25%), and Group C (saline placebo). Postoperative pain was assessed using a visual analog scale (VAS) at 1, 6, 12, and 24 h. Secondary outcomes included rescue diclofenac use, time to first analgesic, total diclofenac dose, time to ambulation, hospital stay, and patient satisfaction. Baseline demographics were comparable across groups. Group B reported the lowest pain scores at 1, 6, and 12 h (0.1 ± 0.3; 2.9 ± 1.1; 2.1 ± 0.3, respectively), followed by Group A, while Group C had the highest (*p* < 0.001). At 24 h, pain scores were similar (*p* = 0.087). Diclofenac requirement was significantly reduced in Group B (26.7%) compared to Groups A (83.3%) and C (100%) (*p* < 0.001). Time to first rescue analgesic was longest in Group B (7.1 ± 1.1 h vs. 2.9 ± 0.7 and 1.5 ± 0.8; *p* < 0.001). Total 24-hour diclofenac consumption was lowest in Group B (75 mg) versus Groups A (114 ± 38.2 mg) and C (142.5 ± 22.9 mg; *p* < 0.001). Early ambulation occurred fastest in Group B (3.5 ± 0.9 h), followed by Group A (5.1 ± 0.6 h) and Group C (6.9 ± 0.8 h; *p* < 0.001). Hospital stay was 24 h in all groups. Patient satisfaction was highest in Group B (7.3 ± 1.2), intermediate in Group A (4.2 ± 1.6), and lowest in Group C (3.0 ± 0.8; *p* < 0.001). Both intraperitoneal and trocar site local anesthetic administration significantly reduced postoperative pain and analgesic requirements compared with placebo. Intraperitoneal administration demonstrated superior efficacy, leading to earlier ambulation and higher patient satisfaction. Routine use of intraperitoneal local anesthetic may enhance postoperative recovery in gynecological laparoscopy.

Trial registration ClinicalTrials.gov Identifier: NCT07030647 (retrospectively registered 20 June 2025).

## Introduction

Laparoscopic gynecological surgery has become the standard approach for many benign and malignant conditions due to its minimally invasive nature, reduced postoperative discomfort, and expedited recovery^1^. Despite these advantages, postoperative pain particularly in the immediate hours following surgery remains a significant concern, often necessitating opioid analgesia, which carries risks of adverse effects such as nausea, sedation, and delayed mobilization^2^.

Intraperitoneal administration of local anesthetics (IPLA) has emerged as a potential strategy to mitigate postoperative pain by targeting peritoneal nociceptors directly^3^. Several studies have explored the efficacy of IPLA in various surgical contexts, including gynecological procedures^4,5^. For instance, a systematic review and meta-analysis by Marks et al. demonstrated that intraperitoneal instillation of local anesthetics significantly decreased pain during the first 6 h after gynecologic laparoscopy^4^. Similarly, a comprehensive review by Boulianne et al. found that IPLA reduced postoperative pain intensity at 6, 12, 24, and 48 h following intra-abdominal surgery, although the certainty of evidence was low^6^. Moreover, Patients who received port-site local anesthetic infiltration demonstrated earlier ambulation times compared to those who did not, highlighting the role of effective pain management in promoting mobility^7^. Additionally, these patients experienced shorter stays in the post-anesthesia care unit (PACU), further indicating a smoother recovery trajectory.

However, the literature presents conflicting evidence regarding the analgesic effect of local anesthetic injection on postoperative pain in gynecological laparoscopic surgeries. These discrepancies highlight the need for further high-quality randomized controlled trials to elucidate the role of IPLA in postoperative pain management.

In this study, we aim to assess the analgesic efficacy of local anesthetic injection on postoperative pain in gynecological laparoscopic surgeries.

## Patients and methods

### Study design and setting

This randomized, double-blind, three arms clinical trial was conducted on 90 women scheduled for elective gynecological laparoscopic surgery between March 2024 and March 2025 at the Department of Obstetrics and Gynecology, Kasr Al-Ainy Hospital, Faculty of Medicine, Cairo University, Cairo, Egypt (ClinicalTrials.gov NCT07030647; retrospectively registered 20 June 2025). All procedures were carried out in accordance with the ethical standards of the Declaration of Helsinki (2013 revision).

### Inclusion criteria

Participants eligible for inclusion in this study were women aged 18 to 60 years who were scheduled to undergo elective gynecological laparoscopic procedures. These procedures included evaluations for primary or secondary infertility, investigations for suspected intrauterine pathology, and assessments for suspected tubal or peritoneal pathology requiring exploration.

### Exclusion criteria

The study excluded participants who met any of the following criteria: a known allergy or hypersensitivity to local anesthetics such as lidocaine or bupivacaine; a body mass index (BMI) of 35 kg/m² or higher, due to the potential impact on surgical outcomes and pain perception; severe hepatic or renal impairment; chronic pain conditions requiring regular opioid use; and a history of major abdominal surgery, which could influence both pain perception and the effectiveness of local anesthetics.

## Methods

The study followed a standardized procedure for all participants. Researchers first obtained informed consent, then conducted a comprehensive clinical assessment, including detailed history taking (demographics, medical and surgical history, allergies, particularly to local anesthetics, chronic pain conditions, and prior analgesic use) with emphasis on hepatic or renal comorbidities. They performed general and gynecological examinations to evaluate vital signs, BMI, and confirm surgical indications. Preoperative investigations included routine laboratory tests (CBC, LFTs, RFTs, coagulation profile, fasting glucose), ECG for participants over 40 or with cardiovascular history, and pelvic ultrasound to verify surgical indications and detect relevant pathology.

This study is a large randomized controlled trial (ClinicalTrials.gov Identifier: NCT07030647) that included three groups and a total of 90 patients comparing the port site local anesthesia group (Group A), intraperitoneal bupivacaine group (Group B), and the placebo control group (Group C).

### Anesthesia protocol

All patients received a standardized general anesthesia protocol. Induction was performed using intravenous propofol (1.5–2.5 mg/kg) and fentanyl (1–2 µg/kg). Neuromuscular blockade was achieved using atracurium at a dose of 0.4–0.5 mg/kg IV. Airway management involved mask ventilation followed by endotracheal intubation, with confirmation of tube placement using end-tidal CO₂ monitoring.

Anesthesia was maintained with isoflurane at a minimum alveolar concentration (MAC) of 1.2% in a 50% oxygen/air mixture. Additional doses of atracurium (0.08–0.1 mg/kg IV) were administered every 15–25 min as needed, based on neuromuscular monitoring. Fentanyl was given as 25–50 µg IV boluses every 30–60 min to ensure adequate analgesia during the procedure. At the end of surgery, neuromuscular blockade was reversed with neostigmine (0.04–0.07 mg/kg IV, max 5 mg) and atropine (0.015–0.02 mg/kg IV) to counteract muscarinic effects. Patients were extubated once fully awake and able to follow commands, with spontaneous ventilation confirmed.

### Surgical procedure and anesthetic administration

This prospective, double-blind randomized controlled trial enrolled 90 patients who were randomly assigned into three groups using a computer-generated sequence. Group A received 5 mL of 0.25% bupivacaine subcutaneously at the trocar sites prior to insertion, followed by 40 mL of saline sprayed intraperitoneally after surgery. Group B received intraperitoneal 0.25% bupivacaine (40 mL) and subcutaneous saline (5 mL), while Group C received saline both intraperitoneally and subcutaneously. Allocation concealment was maintained using sealed opaque envelopes, and both patients and clinicians were blinded to group assignment.

During the surgical procedure, trocar sizes ranging from 5 mm to 10 mm were used. Anesthetic solutions were prepared under sterile conditions. Patients in Group A received 5 ml of 0.25% bupivacaine subcutaneously at the trocar sites before insertion, with 40 ml of saline sprayed intraperitoneally postoperatively. Patients in Group B received 5 ml of saline subcutaneously at the trocar sites preoperatively, while 40 ml of 0.25% bupivacaine was sprayed intraperitoneally postoperatively. The control group (Group C) received 5 ml of saline subcutaneously at the trocar sites preoperatively and 40 ml of saline intraperitoneally postoperatively.

The injection procedure was carefully executed to ensure safety and efficacy. Prior to injection, the skin at each trocar site was disinfected using an antiseptic solution. A fine needle was used for subcutaneous injections, with negative pressure applied beforehand to avoid inadvertent intravascular administration. All solutions were injected slowly to reduce discomfort and ensure even tissue distribution.

For intraperitoneal administration, the prepared solution was instilled at the end of the laparoscopic procedure, immediately before trocar removal. A laparoscopic irrigation-suction cannula was introduced through the primary port, and the solution (either 0.25% bupivacaine or saline, per group allocation) was sprayed evenly across the peritoneal cavity under direct vision. Key anatomical areas including the uterosacral ligaments, pouch of Douglas, anterior and lateral pelvic peritoneum were targeted to ensure maximal analgesic coverage. No suctioning was performed following instillation to preserve contact time between the anesthetic and peritoneal surfaces. The laparoscope was systematically rotated to visually confirm uniform distribution.

### Postoperative analgesia protocol

A uniform postoperative analgesic protocol was followed for study groups. All patients were monitored in the recovery area and inpatient ward. If a patient reported a VAS score greater than 3, intramuscular diclofenac 75 mg was administered as rescue analgesia. No additional analgesics were given within the 24-hour study window, and the need for analgesia, time to first dose, and total diclofenac dose were recorded for each patient.

### Postoperative follow-up

Researchers assessed postoperative pain intensity using the Visual Analog Scale (VAS) at 1, 6, 12, and 24 h after surgery. Patients rated their pain on a validated 0–10 scale, where 0 indicated no pain and 10 represented the worst pain imaginable. In addition to pain scores, investigators recorded the time of the first request for analgesia and the total amount of analgesics administered as secondary outcome measures. Diclofenac Sodium, a nonsteroidal anti-inflammatory drug (NSAID), served as the primary analgesic. At 24 h post-surgery, patients completed a standardized questionnaire to evaluate satisfaction with pain management. The research team also documented the duration of hospital stay for each patient. Preoperative VAS scores were not recorded, as all participants were asymptomatic and not experiencing active pain at the time of surgery.

### Outcomes measurements

Pain intensity was measured using the Visual Analog Scale (VAS) at 1, 6, 12, and 24 h postoperatively. The VAS is a validated 10 cm scale, where 0 indicates no pain and 10 reflects the worst pain imaginable. In addition, the time to first request for analgesia was recorded, defined as the interval between the end of surgery and the patient’s initial request for pain relief. A longer duration was considered indicative of superior analgesic efficacy. Secondary outcomes, including total analgesic consumption were documented, with diclofenac sodium. Reduced analgesic requirements were interpreted as evidence of better pain control. Patient satisfaction with pain management was assessed using a standardized questionnaire administered 24 h after surgery. This evaluation included ratings of pain relief, comfort, and overall satisfaction with postoperative care. Finally, the length of hospital stay was recorded from the end of the surgical procedure until discharge. A shorter duration was considered reflective of more effective pain control and improved recovery. There were no reported intraoperative or postoperative complications in any group, with no significant intergroup differences. In addition, all patients identified as ethnically Egyptian.

### Sample size calculation

Sample size was determined using G*Power version 3.1.9.2, based on previous studies evaluating postoperative VAS scores after laparoscopic surgery^8^. Assuming an effect size of f = 0.35, a minimum of 84 participants (28 per group) was required to achieve 80% power at a significance level of α = 0.05. To ensure equal allocation across groups and strengthen statistical robustness, a total of 90 participants were recruited (30 per group in Groups A, B, and C).

### Statistical analysis

The collected data were coded, tabulated, and analyzed using IBM SPSS Statistics version 22.0 (IBM Corp., Chicago, USA, 2013) and Microsoft Excel 2007. Descriptive statistics were applied to quantitative data as minimum, maximum, mean, and standard deviation for normally distributed variables, and to qualitative data as frequencies and percentages. Inferential statistical analyses included the Shapiro-Wilk test to assess normality. For comparisons between two independent groups with normally distributed quantitative data, the independent t-test was used. Categorical variables were analyzed using the Chi-square test or Fisher’s Exact test when expected cell counts were small. A p-value of less than 0.05 was considered statistically significant.

## Results

**Fig. 1:**
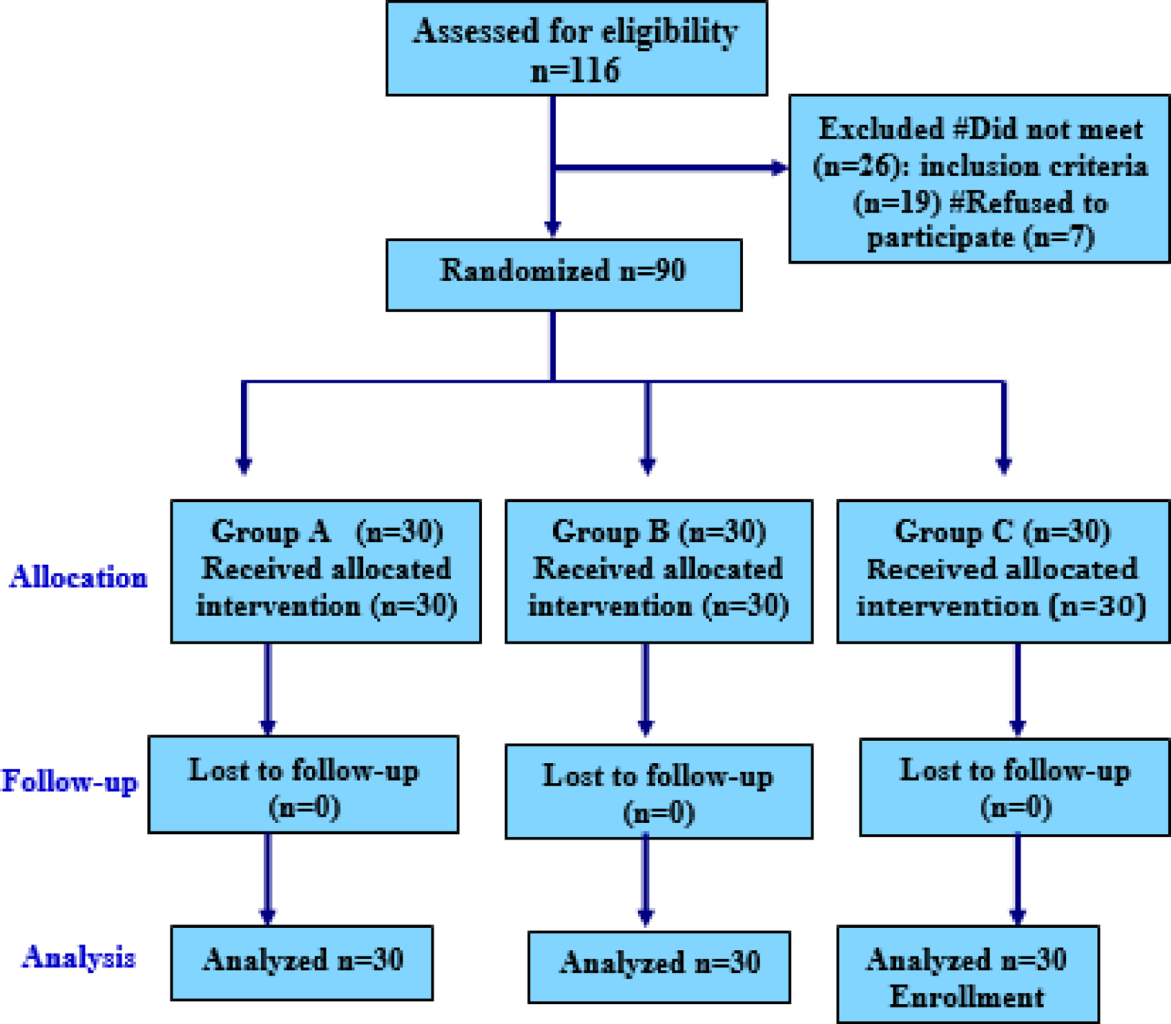
Study flow diagram Group A: Port site local anesthesia injection group. Group B: Intraperitoneal local anesthesia injection group. Group C: Saline Control group.

The study included 90 women **(**Fig. 1**)**.The three study groups were comparable with respect to age, BMI, and operative duration, with no statistically significant differences observed (*p* > 0.05) **(**Table 1**)**.

**Table 1:** Demographic data.

Variable	Group A (*n* = 30) port site LA	Group B (*n* = 30) intraperitoneal LA	Group C (*n* = 30) saline control group	*p*-value
Age (years)	31.9 ± 4.4 (25–43)	32.6 ± 5.1 (24–42)	32.4 ± 5.4 (23–44)	0.866^
BMI (kg/m²)	29.4 ± 3.1 (23.4–34.4)	28.9 ± 2.8 (23.8–34.7)	29.3 ± 2.1 (25.2–33.8)	0.808^
Operation duration (min)	35.3 ± 3.8 (26–42)	34.9 ± 2.9 (29–41)	34.2 ± 4.3 (28–46)	0.554^

^ANOVA test; #Chi-square test. *BMI * Body Mass Index,* LA * local anesthesia. All participants were Egyptian/Middle Eastern.

**Fig. 2:**
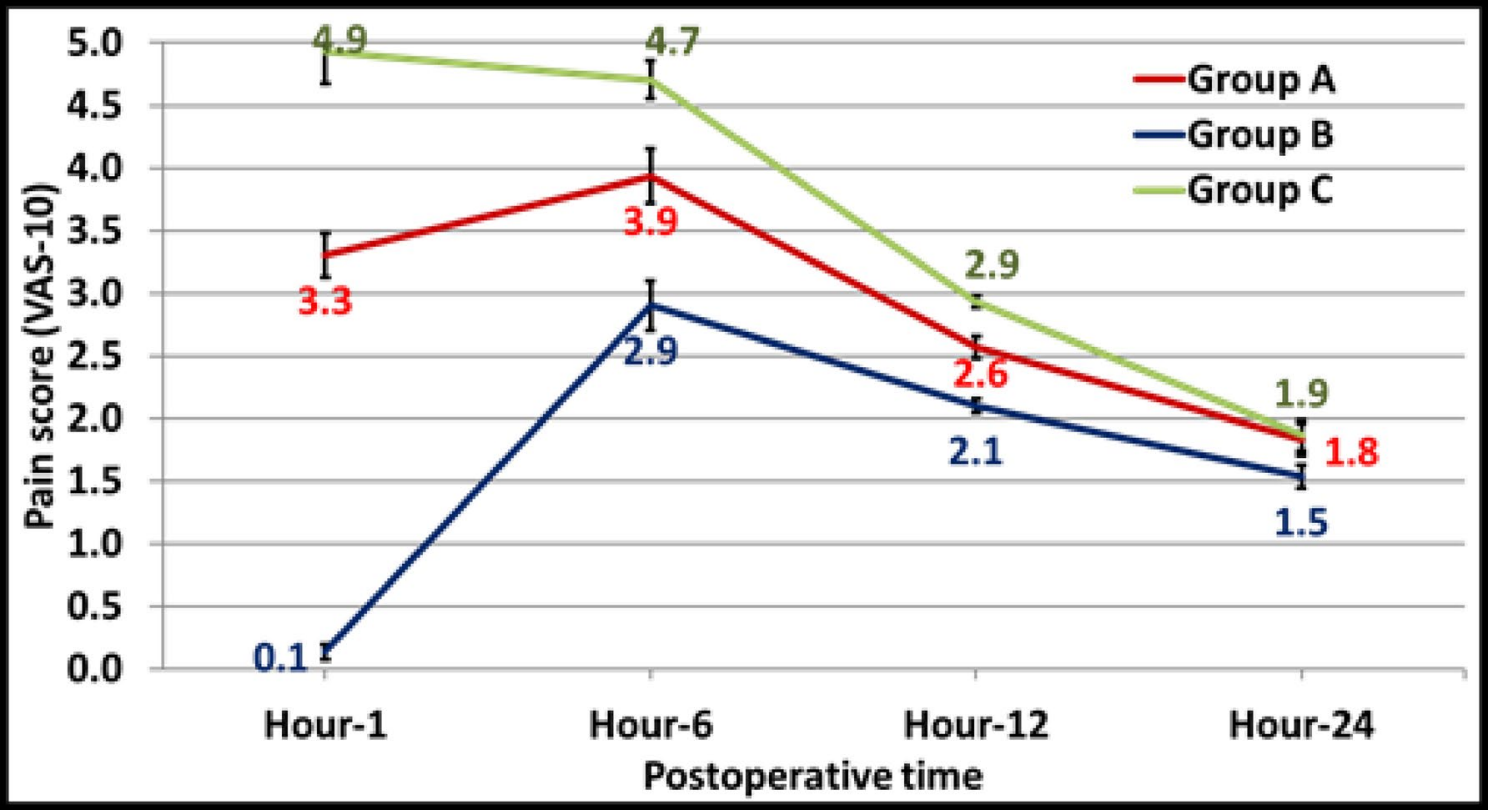
Pain score (VAS-10) between sudy groups. Group A: Port site local anesthesia injection group. Group B: Intraperitoneal local anesthesia injection group. Group C: Saline control group.

Pain scores differed significantly across the groups at 1, 6, and 12 h postoperatively (*p* < 0.001). Group B (intraperitoneal LA) consistently reported the lowest pain scores, while Group C (Saline Control group) had the highest. By 24 h, pain scores decreased in all groups, and the difference was not statistically significant (*p* = 0.087) (Table 2) (Fig. 2**)**.

**Table 2:** Pain score (VAS-10).

Postoperative time	Group A (*n* = 30) port site LA	Group B (*n* = 30) Intraperitoneal LA	Group C (*n* = 30) Saline Control group	*p*-value
Hour-1	3.3 ± 1.0^a^ (3–7)	0.1 ± 0.3^b^ (0–1)	4.9 ± 1.5^c^ (3–7)	< 0.001*
Hour-6	3.9 ± 1.2^a^ (3–6)	2.9 ± 1.1^b^ (2–6)	4.7 ± 0.8^c^ (3–6)	< 0.001*
Hour-12	2.6 ± 0.5^a^ (2–3)	2.1 ± 0.3^b^ (2–3)	2.9 ± 0.3^c^ (2–3)	< 0.001*
Hour-24	1.8 ± 0.7 (1–3)	1.5 ± 0.5 (1–2)	1.9 ± 0.7 (1–3)	0.087

^ANOVA test; LA local anesthesia; *Significant. Homogenous groups had the same symbol “a, b and c” based on post hoc Bonferroni test.

**Fig. 3:**
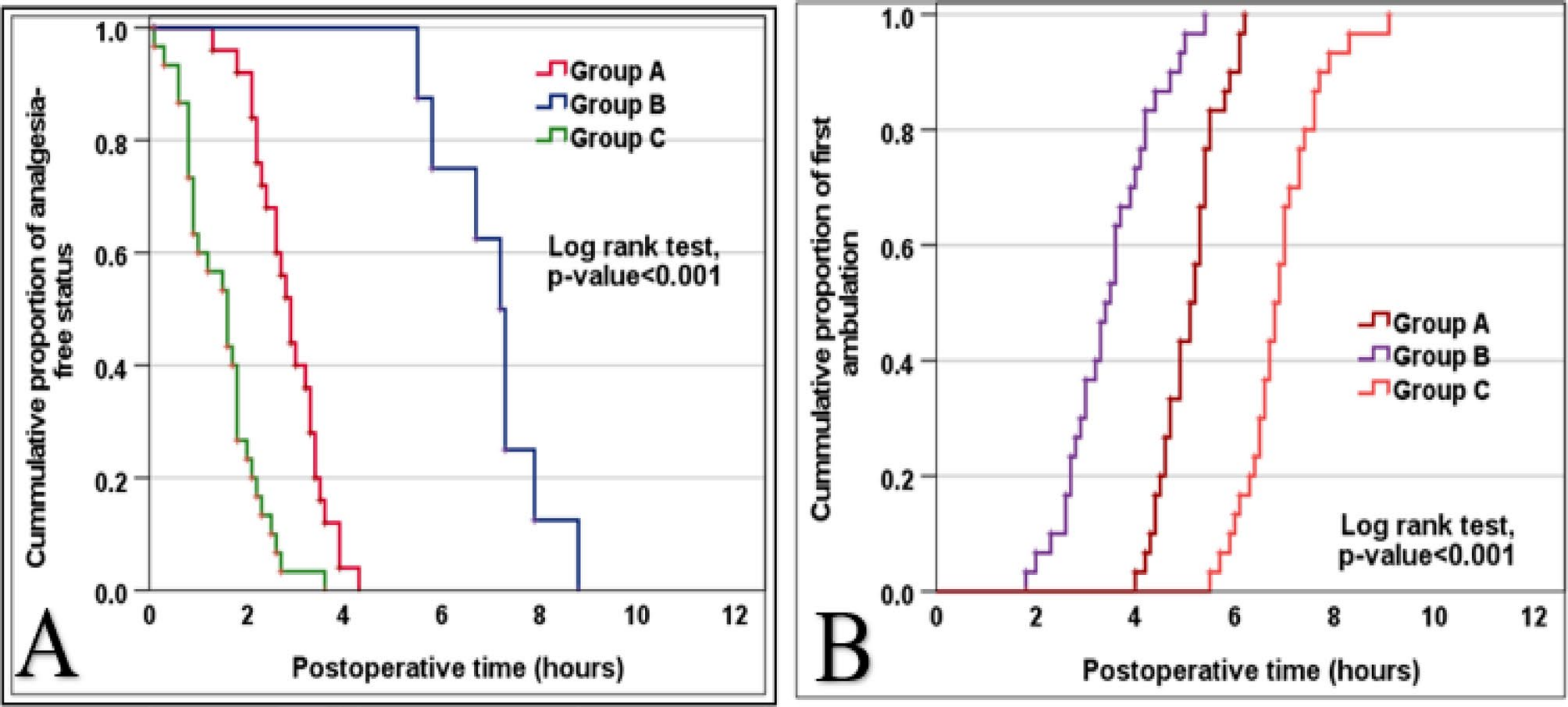
(**A**) Kaplan-Meier curve for rate of first rescue Diclofenac dose between the study groups. (**B**): Kaplan-Meier curve for rate of first ambulation between the study groups Group A: Port site local anesthesia injection group. Group B: Intraperitoneal local anesthesia injection group. Group C: Saline Control group.

The need for diclofenac analgesia varied significantly among groups (*p* < 0.001). Almost all patients in Groups A and C required diclofenac, whereas only one-quarter of Group B did. Moreover, Group B demonstrated a significantly longer time to first analgesic request and the lowest total diclofenac dose compared with the other groups (*p* < 0.001) **(**Table 3**) (**Fig. 3A**)**.

**Table 3:** Diclofenac analgesia requirement.

Variable	Group A (*n* = 30) port site LA	Group B (*n* = 30) intraperitoneal LA	Group C (*n* = 30) saline control group	*p*-value
diclofenac required, n (%)	25 (83.3%)^a^	8 (26.7%)^b^	30 (100.0%)^a^	< 0.001#*
Time to first diclofenac dose (h)	2.9 ± 0.7^a^ (1.3–4.3)	7.1 ± 1.1^b^ (5.5–8.8)	1.5 ± 0.8^a^ (0.1–3.6)	< 0.001^*
Total diclofenac dose in 24 h (mg)	114.0 ± 38.2^a^ (75–150)	75.0 ± 0.0^b^	142.5 ± 22.9^a^ (75–150)	< 0.001^*

^ANOVA test; #Chi-square test; *Significant. Groups with the same superscript letters are not significantly different (Bonferroni post hoc). LA = local anesthesia; All Group B patients requiring analgesia received a single standard dose of 75 mg diclofenac; hence, the SD is 0.0.

Time to first ambulation differed significantly between groups (*p* < 0.001). Group B achieved the earliest ambulation, followed by Group A, while Group C had the longest delay. Hospital stay was uniformly 24 h for all patients (Table 4) (Fig. 3B**)**.

**Table 4:** Time to first Ambulation.

Variable	Group A (*n* = 30) Port site LA	Group B (*n* = 30) Intraperitoneal LA	Group C (*n* = 30) Saline Control group	*p*-value
Time to first ambulation (h)	5.1 ± 0.6^a^ (4.0–6.2)	3.5 ± 0.9^b^ (1.8–5.4)	6.9 ± 0.8^c^ (5.5–9.1)	< 0.001^*

^ANOVA test; LA local anesthesia,*Significant. Homogeneous groups share the same superscript letters (Bonferroni post hoc).

Patient satisfaction scores were significantly higher in Group B compared with Groups A and C (*p* < 0.001). Group C reported the lowest satisfaction levels overall (Table 5).

**Table 5:** Patient Satisfaction.

Variable	Group A (*n* = 30) Port site LA	Group B (*n* = 30) Intraperitoneal LA	Group C (*n* = 30) Saline Control group	*p*-value
Satisfaction score (0–10)	4.2 ± 1.6^a^ (2–7)	7.3 ± 1.2^b^ (4–8)	3.0 ± 0.8^c^ (2–5)	< 0.001^*

^ANOVA test; LA local anesthesia, *Significant. Homogeneous groups share the same superscript letters (Bonferroni post hoc).

## Discussion

In this study, demographic characteristics were comparable across the three groups Group A (port-site local anesthetic injection), Group B (intraperitoneal injection), and Group C (no anesthetic) with no statistically significant differences in age, BMI, or operative time, confirming appropriate randomization (*p* > 0.05).

These findings are in line with previous studies on laparoendoscopic single-site surgery and laparoscopic appendectomy, which also reported no demographic differences across study groups^9,10^. However, some trials reported significant demographic variability^11^, or findings inconsistent with ours, possibly due to differences in surgical type and patient selection^12,13^.

Group B consistently reported the lowest pain scores, particularly at 1, 6, and 12 h, followed by Group A, while Group C experienced the highest pain levels. Intraperitoneal administration therefore provided superior analgesia, with trocar site infiltration offering intermediate benefit. These results concur with prior studies and a meta-analysis demonstrating significant early pain reduction with intraperitoneal local anesthesia^10,14,15^. Conversely, several trials found no significant difference with local anesthetic infiltration^13,16–18^, which may be explained by variations in surgical procedures, anesthetic type, timing, or the inherently mild pain associated with certain operations^9,13,18^.

Group B also required significantly less rescue diclofenac (26.7%) compared to Groups A (83.3%) and C (100%). Time to first dose was longest in Group B (7.1 h), and total 24-hour consumption was lowest, confirming superior efficacy. Similar reductions in analgesic requirements were previously observed with intraperitoneal infiltration^10^, whereas other studies reported no effect of local anesthetics on analgesic use^11,13,17^. Differences likely relate to the anesthetic agent, timing of administration, or type of rescue regimen used, as some trials employed PCA or non-standardized regimens^13,17^. Studies using combined trocar site and intraperitoneal approaches reported outcomes more consistent with ours^10,12^.

Early ambulation, a marker of functional recovery, was achieved earliest in Group B (3.5 h), followed by Group A (5.1 h), and latest in Group C (6.9 h), with significant differences among all groups (*p* < 0.001). These findings demonstrate that intraperitoneal anesthesia accelerates postoperative recovery through superior pain control. While some trials reported no improvement^18,19^, others corroborated our results^10,12^. Discrepancies may be related to surgical type, anesthetic selection, or routine early ambulation protocols that masked differences^9,13^.

Patient satisfaction paralleled pain and recovery outcomes, being highest in Group B (7.3/10), intermediate in Group A (4.2/10), and lowest in Group C (3.0/10). These findings align with reports of higher satisfaction following effective analgesic interventions^12,14,15^. However, other trials observed no significant differences^13,18^, likely influenced by patient expectations, cultural factors, multimodal analgesia use, or variations in anesthetic protocol.

The study’s major strength lies in its three-arm randomized design, directly comparing trocar site versus intraperitoneal administration against a control within the same trial. This design enabled comprehensive evaluation of multiple outcomes, including pain, analgesic use, ambulation, and satisfaction, supported by systematic VAS monitoring.

Limitations include the modest sample size (90 patients) and single-center design, which may restrict generalizability. Pain scores were patient-reported and thus subject to subjective variability. Additionally, the study only assessed outcomes within 24 h, without long-term follow-up, and did not explore varying doses or combinations of anesthetic agents.

## Conclusion

Both intraperitoneal and trocar site local anesthetic administration significantly reduced postoperative pain, lowered analgesic requirements, and facilitated earlier recovery compared to no anesthesia. However, the intraperitoneal approach demonstrated superior efficacy and was associated with greater patient satisfaction, underscoring its value in enhancing overall postoperative comfort and experience.

## Data Availability

The datasets generated and/or analyzed during the current study are available from the corresponding author on reasonable request.

## References

[1] Levy, L. & Tsaltas, J. Recent advances in benign gynecological laparoscopic surgery. *Fac. reviews* **10**, 60 (2021). 10.12703/r/10-60 PMC8361750 34409423

[2] Nurul Amin, M. et al. Postoperative pain management in gynecologic laparoscopic surgeries. *Int. J. Res. Med. Sci.* **13** (1), 102–106 (2024).

[3] Choi, G. J. et al. Effect of intraperitoneal local anesthetic on pain characteristics after laparoscopic cholecystectomy. *World J. Gastroenterol.* **21** (47), 13386 (2015). 26715824 10.3748/wjg.v21.i47.13386 PMC4679773

[4] Marks, J. L., Ata, B. & Tulandi, T. Systematic review and metaanalysis of intraperitoneal instillation of local anesthetics for reduction of pain after gynecologic laparoscopy. *J. Minim. Invasive Gynecol.* **19** (5), 545–553 (2012). 22763313 10.1016/j.jmig.2012.04.002

[5] Mostafa, M. F. & Zahran, K. M. Intraperitoneal Lidocaine versus ketamine for postoperative analgesia after gynecological laparoscopies; randomized controlled clinical study. *MJMR* **27**, 126–136 (2016).

[6] Boulianne, M. et al. Intraperitoneal local anesthetics for postoperative pain management following intra-abdominal surgery: a systematic review and meta-analysis. *BMC Anesthesiol*. **25** (1), 235 (2025). 40348992 10.1186/s12871-025-03105-y PMC12065176

[7] Hossain, M. M. M. et al. Evaluation of the effectiveness of local anesthetic agent infiltration into Port sites at preoperative period with it’s correlation to post operative analgesia in laparoscopic surgical Procedures-A retrospective and comparative study. *SAS J. Surg.* **3**, 361–369 (2025).

[8] Bodian, C. A. et al. The visual analog scale for pain: clinical significance in postoperative patients. *Anesthesiology* **95** (6), 1356–1361 (2001). 11748392 10.1097/00000542-200112000-00013

[9] Seo, J. W. et al. The role of Port site local anesthetic injection in laparoendoscopic single site surgery: a prospective randomized study. *Obstet. Gynecol. Sci.* **63** (3), 387–394 (2020). 32489985 10.5468/ogs.2020.63.3.387 PMC7231930

[10] Čustovic, S., Pandža, H. & Delibegovic, S. Effect of local anesthesia on the postoperative pain after laparoscopic appendectomy. *J. Laparoendosc. Adv. Surg. Tech.* **29** (1), 65–71 (2019). 10.1089/lap.2018.0452 30260724

[11] Joe-Ikechebelu, N. N. et al. A randomized controlled trial on efficacy and safety of trocar-site infiltration with Lidocaine for postoperative pain relief after diagnostic laparoscopy. *Gynecol. Obstet. Invest.* **84** (1), 71–78 (2019). 30145600 10.1159/000490565

[12] Shady, N. W. et al. Effect of intraperitoneal and incisional Port site Lidocaine on pain relief after gynecological laparoscopic surgery: A randomized controlled study. *Middle East. Fertility Soc. J.* **23** (1), 63–67 (2018).

[13] Tam, T. et al. Infiltration of bupivacaine local anesthetic to trocar insertion sites after laparoscopy: a randomized, double-blind, stratified, and controlled trial. *J. Minim. Invasive. Gynecol.* **21** (6), 1015–1021 (2014). 24792311 10.1016/j.jmig.2014.04.013

[14] Hirsch, M., Tariq, L. & Duffy, J. M. Effect of local anesthetics on postoperative pain in patients undergoing gynecologic laparoscopy: A systematic review and Meta-analysis of randomized trials. *J. Minim. Invasive Gynecol.* **28** (10), 1689–1698 (2021). 33991671 10.1016/j.jmig.2021.04.024

[15] Hortu, I. et al. Impact of bupivacaine injection to trocar sites on postoperative pain following laparoscopic hysterectomy: results from a prospective, multicentre, double-blind randomized controlled trial. *Eur. J. Obstet. Gynecol. Reproductive Biology*. **252**, 317–322 (2020). 10.1016/j.ejogrb.2020.07.007 32653604

[16] Netpreeya Nakngam, M., Phornsawan Wasinghon, M. & Auttaya Ratanakaew, M. Bupivacaine local infiltration at trocar insertion sites after gynecologic laparoscopic surgery: A randomized, Double-blind, Placebo-Controlled trial. *J. Med. Assoc. Thai*. **107** (3), 185–190 (2024).

[17] Malik, R. & Verma, R. Does local infiltration of anesthesia reduce Port-site pain in gynecological laparoscopic surgeries?? A pilot study. *Gynecol. Minim. Invasive Therapy*. **13** (2), 101–104 (2024). 10.4103/gmit.gmit_77_22 PMC11192289 38911315

[18] Gluck, O. et al. The effect of subcutaneous and intraperitoneal anesthesia on post laparoscopic pain: a randomized controlled trial. *Sci. Rep.* **11** (1), 81 (2021). 33420214 10.1038/s41598-020-80130-6 PMC7794319

[19] Lysander, S. S. et al. Comparison of trocar site versus trocar site plus intraperitoneal instillation of local anesthetic for shoulder pain following laparoscopic abdominal surgery. *Anesth. Essays Res.* **15** (4), 375–378 (2021). 35422541 10.4103/aer.aer_156_21 PMC9004265

